# Supplementary material for: The Role of Chemokines and Small Leucine-Rich Proteoglycans in Cardiac Remodeling in Immunosuppressant-Treated Male Rats
Source: Int J Mol Sci. 2025 Jul 3;26(13):6414. doi: 10.3390/ijms26136414 (PMC12249708; doi:10.3390/ijms26136414)
Supplement: Supplementary file 1 [file ijms-26-06414-s001.zip › ijms-3593910-supplementary.pdf]

Table S1. Mean results of CXCR5, CXCL13 and fibromodulin with 95% confidence intervals (CI) in control and experimental groups. Values are presented as: mean (95% CI).

Group	CXCR5	CXCL13	Fibromodulin
C	0.897 (0.65-1.14)	468.406 (359.11-577.7)	14.531 (13.34-15.72)
TRG	0.946 (0.7-1.19)	267.284 (126.18-408.39)	15.334 (13.12-17.55)
CRG	1.054 (0.77-1.34)	354.351 (273.01-435.7)	13.47 (12.74-14.2)
MRG	1.185 (0.68-1.69)	375.844 (293.31-458.38)	13.785 (10.55-17.02)
CMG	1.734 (1.22-2.25)	753.074 (545.15-961)	22.872 (19.45-26.3)
TMG	1.754 (1.17-2.33)	565.798 (399.66-731.93)	20.364 (16.99-23.74)
